# From microbiota toward gastro-enteropancreatic neuroendocrine neoplasms: Are we on the highway to hell?

**DOI:** 10.1007/s11154-020-09589-y

**Published:** 2020-09-15

**Authors:** Giovanni Vitale, Alessandra Dicitore, Luigi Barrea, Emilia Sbardella, Paola Razzore, Severo Campione, Antongiulio Faggiano, Annamaria Colao, on behalf of NIKE, Manuela Albertelli, Barbara Altieri, Filomena Bottiglieri, Federica De Cicco, Sergio Di Molfetta, Giuseppe Fanciulli, Tiziana Feola, Diego Ferone, Francesco Ferraù, Marco Gallo, Elisa Giannetta, Federica Grillo, Erika Grossrubatscher, Elia Guadagno, Valentina Guarnotta, Andrea M. Isidori, Andrea Lania, Andrea Lenzi, Fabio Lo Calzo, Pasquale Malandrino, Erika Messina, Roberta Modica, Giovanna Muscogiuri, Luca Pes, Genoveffa Pizza, Riccardo Pofi, Giulia Puliani, Carmen Rainone, Laura Rizza, Manila Rubino, Rosa Maria Ruggieri, Franz Sesti, Mary Anna Venneri, Maria Chiara Zatelli

**Affiliations:** 1 Istituto Auxologico Italiano IRCCS, Laboratory of Geriatric and Oncologic Neuroendocrinology Research, Cusano Milanino, MI Italy; 2 Department of Clinical Sciences and Community Health (DISCCO), University of Milan, Milan, Italy; 3 Department of Clinical Medicine and Surgery, University of Naples Federico II, Naples, Italy; 4 Department of Experimental Medicine, Sapienza University of Rome, Rome, Italy; 5 Endocrinology Unit, A.O. Ordine Mauriziano, Turin, Italy; 6 Pathology Department, Cardarelli Hospital, Naples, Italy

**Keywords:** Neuroendocrine tumors, Microbiota, Inflammation, Tumor microenvironment, Cytokines

## Abstract

Gut microbiota is represented by different microorganisms that colonize the intestinal tract, mostly the large intestine, such as bacteria, fungi, archaea and viruses. The gut microbial balance has a key role in several functions. It modulates the host’s metabolism, maintains the gut barrier integrity, participates in the xenobiotics and drug metabolism, and acts as protection against gastro-intestinal pathogens through the host’s immune system modulation. The impaired gut microbiota, called dysbiosis, may be the result of an imbalance in this equilibrium and is linked with different diseases, including cancer. While most of the studies have focused on the association between microbiota and gastrointestinal adenocarcinomas, very little is known about gastroenteropancreatic (GEP) neuroendocrine neoplasms (NENs). In this review, we provide an overview concerning the complex interplay between gut microbiota and GEP NENs, focusing on the potential role in tumorigenesis and progression in these tumors.

## Introduction

In the recent years, several studies have reported the central role of gut microbiota as key determinants of numerous pathologic conditions, including cancer [1–5]. Gut microbiota is represented by different microorganisms that colonize the intestinal tract, mostly the large intestine, such as bacteria, fungi, archaea and viruses [6]. In particular, *Firmicutes* and *Bacteroidetes phyla* are the highly represented ones [7]. Several bacterial species are involved in carcinogenesis. Elevated levels of DNA of Fusobacterium nucleatum have been detected in tumor cells of colorectal adenoma and cancer [8, 9]. In contrast, probiotic bacterium species, including Bifidobacterium and Lactobacillus genera, may exert a protective impact against cancer [10].

**Fig. 1:**
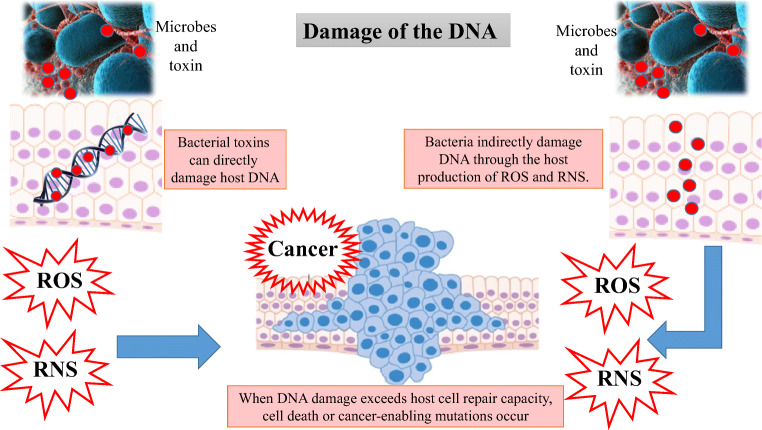
Gut microorganisms can alter the resistance to cell death, and proliferative signalling, by affecting genomic stability, damaging the DNA, and through a microbial competition with others microorganisms. These mechanisms can contribute to carcinogenesis through the increase in mutational events

**Fig. 2:**
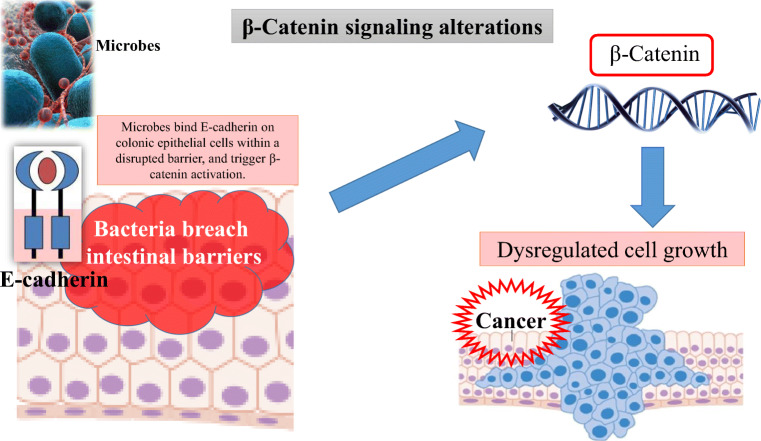
An important target of cancer-associated microbes is the β-catenin signalling. The microbes bind E-cadherin on colonic epithelial cells within a disrupted barrier, and trigger β-catenin activation, resulting in dysregulated cell growth

**Fig. 3:**
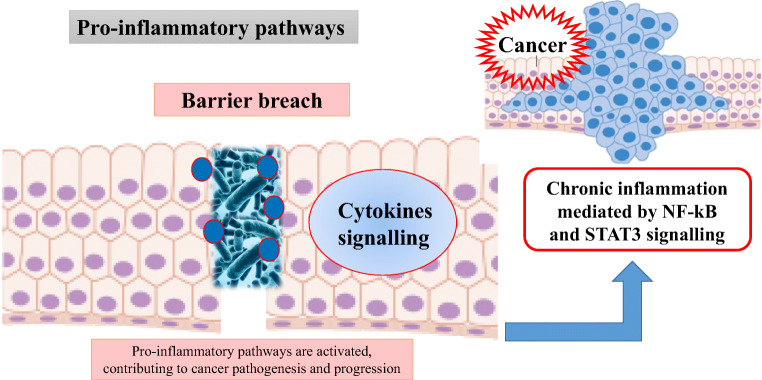
The loss of boundaries between host and microbe and the activation of chronic inflammation via NF-kB and STAT3 signalling promote carcinogenesis

The gut microbial balance has a key role in several functions. Indeed, it modulates the host’s metabolism, maintains the gut barrier integrity, participates in the xenobiotics and drug metabolism, and acts as protection against gastro-intestinal pathogens through the host’s immune system modulation [11–13]. The impaired gut microbiota, called dysbiosis, may be the result of an imbalance in this equilibrium and is linked with the development of tumors [5, 14]. Gut microbiota can interact with the tumor microenvironment, influencing the tumor growth and progression [15, 16]. On the contrary, gut microbiota can act in the detoxification of dietary components and reduction of chronic inflammation [17]. Through this complex crosstalk the gut microbiota, depending on its own composition, may affect the cancer genesis and development, either in a positive or in a negative way. In this context, the gut microbiota can contribute to carcinogenesis through *alteration of the balance of host cell proliferation and death* (Figs. 1 and 2), and the *modulation of immune system function* (Fig. 3) [17**]**.

Gut microorganisms can alter the *resistance to cell death and proliferative signalling of host cells*, by affecting genomic stability, damaging the DNA, and indirectly through a change in indigenous microbiota [18, 19]. These mechanisms can contribute to carcinogenesis through the increase in mutational events (Fig. 1). An example is provided by colibactin, a molecule expressed by *Escherichia coli* [20] and Enterobacteriaceae [21], associated with colorectal carcinogenesis [22, 23]. This molecule causes DNA damage and mutations directly or via production of high levels of reactive oxygen species [18, 24]. Similarly, in colonic epithelial cells, the *Bacteroides fragilis* toxin, through the over production of reactive oxygen and nitrogen species, causes indirectly DNA damage, leading to cell death or cancer-enabling mutations (Fig. 1) [9, 24].

Several microorganisms have proteins that engage host pathways involved in carcinogenesis, such as Wnt/β-catenin pathway (Fig. 2) [25]. The Wnt/β-catenin pathway regulates different cell behaviours [26, 27], such as axis formation during development [28], maintenance of stem cell in adulthood [29], and in tissue regeneration [30]. In the gastrointestinal tract the Wnt pathway maintains the self-renewal capacity of epithelial stem cells and aberrant activation of this pathway may lead to cancer [31]. Therefore, the barrier maintenance between host and microbe represents a critical point in the development of some tumors [32–39]**.**

When the barriers are breached, microbes can act on immune responses leading to *activation pro-inflammatory or immunosuppressive pathway* (Fig. 3). Interestingly, the microbial dysbiosis may contribute to both cancer pathogenesis and progression [13, 40]. In fact, several inflammatory factors such as pro-inflammatory cytokines and chemokines, reactive oxygen and nitrogen species, are associated to growth and spread of the cancer (Fig. 3). Recognition of microbial components, through Toll-like receptors, can activate downstream signalling pathways, such as NF-kB, which leads to the production of proinflammatory factors (Fig. 3) [41]. In addition, the activation of innate immune system due to the breached of gut barriers, leads to adaptive immune responses modulated by several cytokines and STAT3 activation, all associated to cancer progression (Fig. 3) [42–48].

While most of the studies have focused on the association between microbiota and gastrointestinal adenocarcinomas, very little is known about gastroenteropancreatic (GEP) neuroendocrine neoplasms (NENs). In this review, we provide an overview concerning the complex interplay between gut microbiota and GEP NENs, focusing on the potential role in tumorigenesis and progression of these tumors.

## Helicobacter pylori (HP) and NENs: Preclinical and clinical studies

HP is a gram-negative bacterium that infects human beings colonizing gastric mucosa and thus eliciting chronic gastritis [49]. This process can progress within years and decades to chronic atrophic gastritis, that is characterized by a loss of appropriate glands, either in form of lamina propria fibrosis or glandular metaplasia. This condition appears to be a major cause of gastric adenocarcinoma [50–53]. In 1994 the World Health Organization and International Agency for Research on Cancer consensus group reported that epidemiologic and histologic evidences were sufficient to consider HP as a definite carcinogen [54, 55]. Gastric precancerous cascade is determined by both inflammatory process and DNA damage in cells infected by HP (Fig. 1).

Gastric NENs, also defined gastric carcinoids, are rare tumors of the stomach that arise from the enterochromaffin-like (ECL) cells [56]. Several evidences suggested a potential carcinogenic role for HP in NENs.

There are three types of gastric NENs, classified according to their histology and malignant potential. A majority are defined as *type I* that are associated to chronic atrophic gastritis, either autoimmune-driven or as a consequence of HP infection, and *type II*, associated to gastrinoma in patients with Multiple Endocrine Neoplasia Syndromes type 1 syndrome. These first two types are tumors that develop secondary to high gastrin levels. The majority of types I and II gastric carcinoids are small (1–2 cm), multiple, and mainly confined to the gastric mucosa/submucosa layers. These lesions generally have an indolent course associated with low metastatic potential. On the contrary, *type III* gastric carcinoids are solitary and large (>2 cm) tumors, with no known correlation to gastrin production, that infiltrate the muscular layers and are related with the development of local and distant metastases [57, 58].

A longstanding HP infection was shown to be associated with chronic atrophic gastritis [59] and abnormalities in the gastric secretion. Chronic gastritis due to HP has been considered as a risk factor for the development of gastric adenocarcinoma [59]. However, few studies showed that HP infection induces formation of carcinoids of the stomach in animals and humans [58, 60, 61].

In 1999 Hirayama and Colleagues [60] showed that long-term colonization by HP is a crucial risk factor for the development of gastric adenocarcinoma and carcinoid in a Mongolian gerbil model. Kagawa et al. followed the histological changes of HP-infected stomachs of Mongolian gerbils compared to uninfected animals for 24 months and reported that HP infection can cause ECL-like cell tumors due to hypergastrinemia [62]. Interestingly, HP eradication prevented the occurrence of gastric carcinoid in the Mongolian gerbil stomach [63].

In humans, a population-based case-control study, comparing 1,138,390 cancer cases with 100,000 matched individuals without cancer, showed that subjects with chronic atrophic gastritis and pernicious anemia have a significantly increased risk of type I gastric carcinoids (odds ratio, 11.43; 95% CI 8.90–14.69) [64].

Solcia et al. showed a series of 60 gastric endocrine tumors, comprising 44 body-fundus argyrophil carcinoids, of which 23 developed in a background of hypergastrinemia and type A chronic atrophic gastritis (A-CAG), especially characterized with histologic patterns of an autoimmune process. Only 22% of 36 carcinoids and 21% of 19 A-CAG carcinoids had HP colonization, compared to 50% of 14 A-CAG-associated neuroendocrine carcinomas or mixed endocrine-exocrine tumors. On the other hand, 84% of 150 patients with early gastric cancer (*p* < 0.001 versus carcinoids), mostly with A-CAG, had HP colonization [65]**.** They concluded that high gastrin levels and local mechanisms activated by chronic autoimmune gastritis are some of the factors that contribute in the pathogenesis of relatively indolent A-CAG-associated carcinoids, while active end-stage HP gastritis associated to environmental factors could contribute to more severe epithelial transformation, leading to gastric cancer and to neuroendocrine carcinomas or mixed endocrine-exocrine tumors [65]. In addition, most patients with A-CAG, despite having a low incidence of current overt infection, have been previously infected with HP, as demonstrated by the presence of HP antibodies [66].

In a recent study from an Indian NEN Center, gastric carcinoids constituted about 32% of all GEP NENs. At the histopathological review, a high incidence of multifocal atrophy in the antrum, fundus and body was observed, while in autoimmune gastritis, atrophy is especially localized to the gastric body [67]. The authors have speculated that in India, where HP infection is very common, multifocal atrophic gastritis caused by HP can represent a crucial risk factor in the development of gastric NENs.

## Effects of HP colonization on signalling pathways involved in NEN transformation

Although further epidemiological studies are necessary to confirm the association between HP infection and the development of gastric NENs, there are several conceptual evidences of mechanisms involved in these events.

**Fig. 4:**
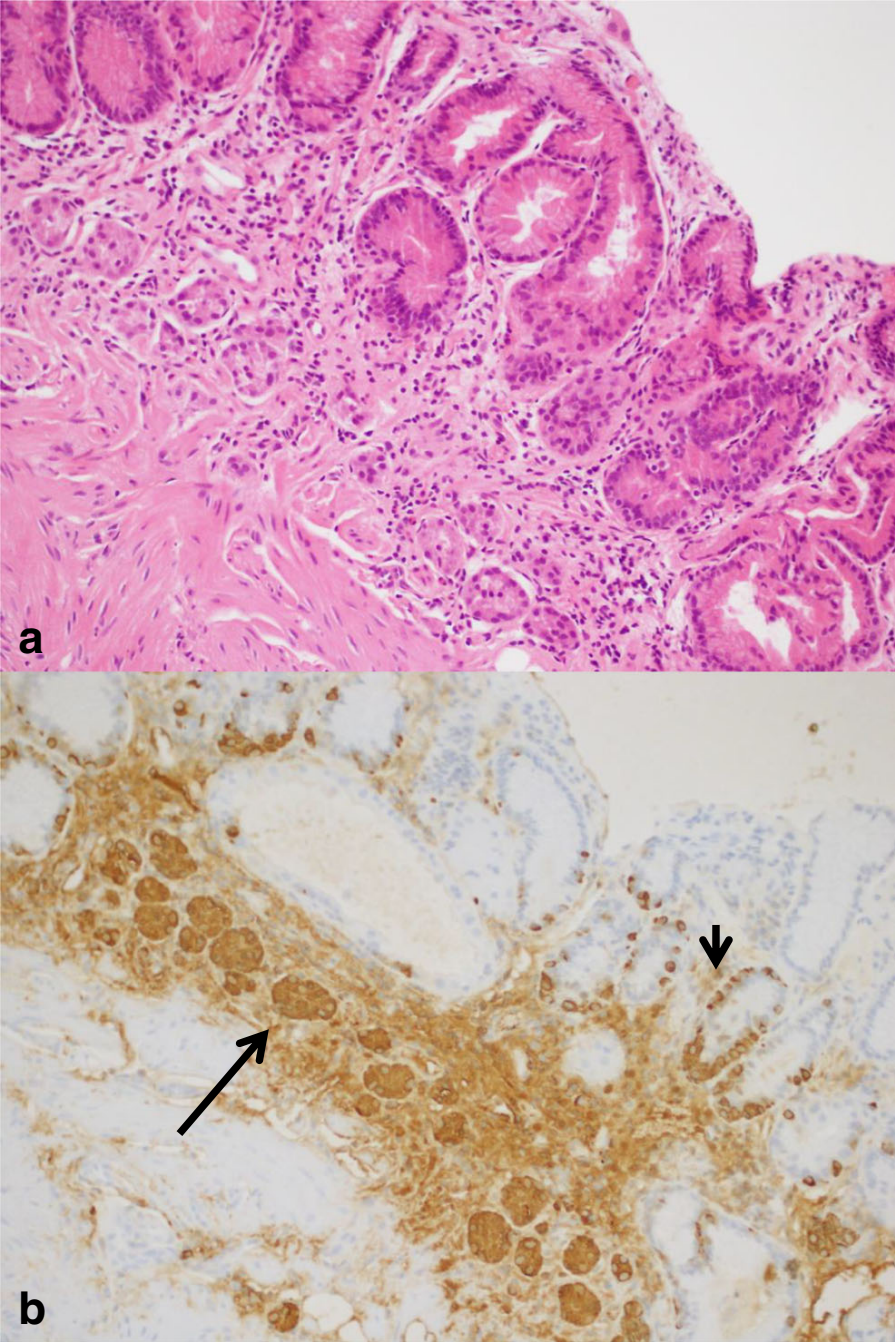
Helicobacter pylori–associated atrophic gastritis. A complete loss of oxyntic glands is evident (**a**) (haematoxylin eosin, 200x magnification) with a linear (short arrow) and nodular (long arrow) ECL cell neuroendocrine hyperplasia (**b**) (immunohistochemistry Chromogranin A, 200x magnification, in an adjacent section of A)

It is clear that HP infection induces the development of inflammatory disorders. Atrophic gastritis and ECL hyperplasia are the final consequence of this inflammatory process (Fig. 4). The destruction of the gastric parietal cells reduces the production of hydrochloric acid, promoting hypergastrinemia. The gastrin excess stimulates not only histamine secretion but also ECL cells proliferation, via the gastrin/ cholecystokinin-B receptor [68]. This process, along with dysplastic lesions, ultimately may lead to the development of NENs [69]. In addition, HP facilitates gastric ECL cell proliferation by other mechanisms. The mucosal inflammation, induced by HP, has been shown to cause excessive apoptosis, which in turn leads to proliferation. Lipopolysaccharides also appear to influence tumor ECL cell proliferation [70]. In rats HP lipopolysaccharides stimulate histamine release via the CD14 receptor. Histamine is a potent mitogenic factor, able to potentiate gastrin-driven DNA synthesis in ECL cells [71]. Another factor involved in ECL cell proliferation is REG protein, a growth factor, which may be stimulated by HP infection [72].

**Fig. 5:**
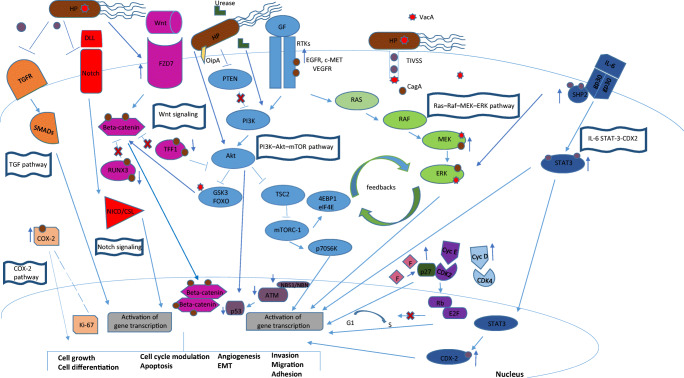
Common cellular signalling pathways involved in GEP NENs and perturbated after HP colonization

In the last years, several molecular pathways have emerged to explain how HP evades host defences, damages gastric tissue and promote tumorigenesis. Three major virulence factors appear to have a role in gastric tumorigenesis: vacuolating cytotoxin A, type IV secretion system, and cytotoxin-associated gene A protein [73]. Although most studies have focused on gastric adenocarcinoma and epithelial cells, in this section we will discuss the intracellular signalling pathways able to disrupt normal physiology of gastrointestinal mucosal cells during HP infection and are in common with the development of GEP NENs (Fig. 5). For most of these HP-perturbated signalling pathways, we cannot exclude a direct involvement also in ECL cells.

### Tyrosine kinase-mediated signalling

Receptor tyrosine kinases belong to a family of receptors that mediate cellular responses to extracellular signals such as growth factors, hormones and cytokines. These receptors play an important role in cell proliferation, survival and differentiation. Several receptor tyrosine kinases are frequently upregulated in GEP NENs, such as the receptor families for vascular endothelial growth factor, epidermal growth factor and tyrosine-protein kinase c-Met [74–77]. Receptor tyrosine kinases activity results in the activation of several transduction systems, including the canonical Ras signalling pathway and PI3K–Akt–mTOR [78].

HP stimulated vascular endothelial growth factor receptor expression in human microvascular endothelial cells (HMEC-1) [79] and epidermal growth factor receptor expression in gastric epithelial cells [80]. HP strains carrying the type IV secretion system induce gastrin promoter activity via epidermal growth factor receptor [81]. Epidermal growth factor receptor is also activated by HP-integrin-β1 interaction [82]. HP infection may activate c-Met expression through cytotoxin-associated gene A protein in gastric epithelial cells, resulting in ERK1, 2 activation [83].

### RAS–RAF–MEK–ERK pathway

The RAS-RAF-MEK-ERK pathway, activated by several growth factors, is involved in cell growth and cell differentiation. Dysregulation of this crucial pathway occurs due to overexpression and/or overactivation of the *RAS* and *RAF* genes [84]. Mutations of *RAS* [85–87] are very rare events in GEP NENs, with reported mutation frequencies [87] of *HRAS* 1% (2/150), *KRAS* 8% (10/125), *NRAS* 0.7% (2/274) or *BRAF* 1% (4/369). Although activating mutations of *BRAF* are rare in GEP NENs [88], wildtype *BRAF* and its activating small G protein, *RAP-1*, are highly prevalent in the majority of GEP NENs. Overexpression of *RAP-1* is able to activate MAPK-signalling and the expression of mitogenic transcription factors of GEP NEN cells [89]. Some authors have reported that RAF-1 signalling cascade activation is associated with the modulation of neuroendocrine phenotype in BON-1 cells, a well-known neuroendocrine tumor cell line [90, 91].

Gastrin, via the cholecystokinin 2 receptor, is the principal regulator of ECL cell proliferation via a MAPK-activated signal transduction cascade [92] and induction of the activator protein-1 complex transcription factor [93]. The latter regulate several genes involved in cell cycle progression (eg, cyclin genes) [94]. ECL cell proliferation is associated with fos/jun transcription activation by the MAPK pathway after gastrin-mediated RAS activation [95].

HP rapidly activates MAPKs upon contact with gastric epithelial cells [96] and, indirectly, promotes gastrin-induced MAPK transduction pathways in ECL cells through histamine release [95]. Several bacterial factors are involved in MAPK activation, including the vacuolating cytotoxin A [97, 98] and cytotoxin-associated gene A protein [99]. However, the type IV secretion system appears to be crucial for complete phosphorylation of ERK and MAPK [96, 100].

### PI3K–AKT–mTOR pathway

The PI3K–AKT–mTOR pathway plays a relevant role in the pathogenesis and progression of GEP NENs [101]. AKT is the major kinase, which regulates cell survival and proliferation by inhibiting proapoptotic mitochondrial proteins and cell-cycle modulators. Dysregulation of this pathway is due to activation of PI3K or loss-of-function mutation of *PTEN*, *TSC2* and *GSK3*. Studies in small bowel NEN cell lines revealed a stronger activation of PI3K/AKT/mTOR pathway compared to that observed in normal ECL cells [102].

In gastric epithelial cells HP infection induced PTEN phosphorylation, which activated AKT [103] and inhibited apoptosis [104]. Interestingly, the HP urease seems to have a relevant role in the activation of the PI3K-AKT-mTOR pathway in gastric cells [105].

### Notch signalling

Notch signalling pathway plays an important role in maintaining a dynamic balance between cell proliferation, differentiation and apoptosis and is an essential signalling in the regulation of inflammatory and immune responses. Notch signalling can have an oncogenic or tumour suppressor role. Histo-pathological studies have shown that Notch-1 is absent or poorly expressed in well-differentiated GEP NENs, suggesting a possible role as a tumour suppressor gene in these tumors [106].

After HP invasion, a significant reduction in the mRNA expression level of Notch-1 and Notch-2, together with low levels of active forms of Notch-1 and Notch-2, have been observed in GES-1, a human gastric epithelial cell line [107].

### Wnt/β-catenin pathway

Wnt/β-catenin pathway is crucial to embryo development and adult tissue homeostasis. Aberrant activation of this pathway can cause uncontrolled cell growth and malignant transformation (Fig. 2). In NENs, cytoplasmic and nuclear beta-catenin accumulation, suggestive of Wnt/β-catenin signalling activation, has been reported in 1/12 gastro-intestinal NENs and 1/6 bronchial carcinoids [108]. Sun and colleagues found cytoplasmic accumulation and/or nuclear translocation of β-catenin in about 30% of gastro-intestinal NENs (27/80) [109]. In 72 cases of gastrointestinal NENs, accumulation of β-catenin in the cytoplasm and/or nucleus has been observed in 79% of cases (57/72) and mutations in exon 3 of β-catenin in 37% of tumors [110]. *APC* gene is a negative regulator that controls β-catenin concentrations and modulates cell adhesion. In ileal NENs, the *APC* gene was deleted in 15% (4/27) and somatic mutations of this gene were detected in 23% (7/30) of examined tumour samples, including 57% missense and 14% nonsense/frameshift mutations [111].

HP activates Wnt/β-catenin signalling through several mechanisms in gastric cells. Cytotoxin-associated gene A protein induces nuclear β-catenin accumulation *in vivo* and *in vitro* [112, 113] and activates the β-catenin through an independent phosphorylation manner in human gastric cancer epithelial cell lines or in rodent gastric cells [114]. The vacuolating cytotoxin A induces Wnt/β-catenin signalling through the activation of PI3K/AKT pathway [115]. HP can also activate Wnt/β-catenin pathway by recruiting tumor-associated macrophages [116]. Wnt/β-catenin activation in HP infection has been linked to angiogenesis in the gastric mucosa, which is an important process for tumorigenesis [117].

### The transforming growth factor-beta (TGF-β) signalling

The TGF-β exists in at least three isoforms: TGF-β1, TGF-β2, and TGF-β3 [118]. TGF-β signalling is mediated by TGF-β receptors 1 and 2 and intracellular SMAD proteins. These factors are involved in cell cycle regulation, apoptosis, tumor angiogenesis and invasion [119–121]. A high expression level of TGF-β receptor 1 (intensity scores 2 and 3) has been detected in almost 100% of GEP NENs [122]. The tumor suppressor *SMAD4* has been demonstrated to be often mutated or deleted in small intestinal NENs in approximately 45% of cases (22/48) [123, 124].

TGF-β1 is a potent stimulator of ECL cell proliferation through downregulation of SMAD4 and activation of the ERK1/2 pathway [125]. *In vivo* studies have shown that HP infection induced upregulation of TGF-β1 in gastric mucosa. This effect is positively correlated with the vacuolating cytotoxin A genotype and the grade of chronic inflammation [126].

### TP53

The *TP53* gene encodes p53, an important tumour suppressor modulating a network of genes implicated in DNA repair, cell growth arrest or cell senescence, apoptosis and autophagy [127]. The main effectors of *TP53* expression are *WIP1*, *MDM2*, *MDMX*, *ATM* and *ATR* genes [128, 129]. Mutations in the *TP53* gene have been consistently detected in poorly differentiated GEP NENs, with a frequency ranging from 20% to 73% of cases [130] and correlate with poor survival [131]. Hu and co-workers [132] observed a high rate of copy number gains of *MDM2* in 22%, *MDM4* in 40% and *WIP1* in 51% of pancreatic NENs. High ATM expression in pancreatic NENs was associated with higher tumour differentiation, lower tumour size, lower recurrence rate and better prognosis [133], while loss of ATM expression was common in metastasized disease and resulted to be associated with a worse prognosis [134]. Interestingly, in the African rodent mastomys *Tp53* seems to have a relevant role during the development of hypergastrinemia-induced ECLoma [135].

HP is able to inhibit the tumor suppressor *TP53* through AKT activation and subsequent degradation of p53 in gastric epithelial cells [136]. Inhibition of p53 may provide advantages to HP and allow it to alter cellular homeostasis without triggering cell cycle arrest or apoptosis [137].

### Cyclin-dependent kinases (CDKs)

The family of CDKs belongs to a superfamily of 20 members, which catalyse the phosphorylation of key proteins and transcription factors implicated in cell cycle transition [138–140]. Cyclin C/CDK3, Cyclin D/CDK4 and Cyclin D/CDK6 are involved in G_0_–G_1_ transition and the early G_1_ phase by phosphorylating the tumour suppressor retinoblastoma protein and thus activating E2F [141]. These pathways are commonly altered in tumors, including GEP NENs [142, 143]. In 92 tumour samples of human pancreatic NENs, overexpression of CDK4 and retinoblastoma protein was detected in 58% and 68%, respectively. Gene amplifications of *CDK4* or *CDK6* were found in 19% (5/26) of investigated pancreatic NENs [144]. p27 is CDK inhibitor encoded by the *CDKN1B* gene and regulates the transition from cell cycle phase G_0_/G_1_ to S and is implicated also in cellular motility and apoptosis. Frameshift mutations or deletions of *CDKN1B* were reported in about 8–23% of small intestinal NENs [145, 146]. Loss of p27 protein expression, which occurred in 21% of 327 GEP NENs, was a predictor of poor prognosis [147]. The inactivation of *RB1* gene, which occurs mainly by somatic mutations, has been reported in 71% of poorly differentiated pancreatic neuroendocrine carcinomas [148]. Both message and protein levels of cyclin D1 increased *in vitro* during ECL cell tumorigenesis [149, 150].

HP and related inflammatory response are associated *in vivo* with alterations in expression of cyclin D1 and CDKN1B and abnormalities in epithelial cell proliferation, cell cycle progression and apoptosis. HP infection can stimulate proliferation of gastric mucosal epithelial cells [151], through activating the MAPK pathway and promoting the expression of cyclin D1 [152]. In addition, HP decreases p27 expression in gastric cells through epigenetic mechanisms [153, 154]. Interestingly, low gastric p27 may promote carcinogenesis associated with HP infection by inhibiting apoptotic pathways [155].

### Interleukin-6/STAT3/CDX2

The interleukin-6/STAT3/CDX2 pathway represents a relevant factor for the tumor progression of gastrointestinal NENs [156–158].

It has been reported that the proinflammatory cytokine interleukin-6 is upregulated during HP infection in the gastric mucosa, with a potential involvement in gastrointestinal tumor development [159]. Interleukin-6 binds to the α-subunit of its specific receptor and activates two main signalling pathways: SHP-2/ERK and JAK/STAT [99], able to promote mucosal inflammation and carcinogenesis [160–163]. In addition, HP infection induces CDX2 expression in patients with chronic gastritis and intestinal metaplasia [163].

### Cyclooxygenase-2 (COX-2)

COX-2 is able to modulate cell apoptosis and adhesion and promote tumor cell metastasis [164]. COX-2 overexpression was observed in 54% (126 of 234) of GEP NENs and was found to be positively correlated with Ki-67 labelling index and associated with poor prognosis [165].

COX-2 is induced in HP–positive gastritis and present at high levels in gastric antrum, where bacterial density is elevated [166]. This suggested that expression of COX-2 was a direct response to HP infection [167]. In patients with HP-positive gastric mucosal lesions, positive detection rate of COX-2 resulted significantly higher than that in HP-negative gastric mucosal lesions [168].

## Inflammatory bowel disease (IBD), NENs and microbiota, is there a possible link?

### IBD and NENs

IBD is a group of inflammatory conditions of the colon and small intestine, that includes Crohn’s disease (CD) and ulcerative colitis.

Data from population study and large pathological and disease registry suggest an association between IBD and intestinal NENs. Among a cohort of 20,917 patients affected by CD, 9 NENs were observed, resulting, in a 7-fold increased neoplastic risk, as compared to the general population [169]. In a prospective observational 7-year follow-up cohort study in 590 patients with mono-institutional IBD diagnosis, neuroendocrine carcinoma and rectal carcinoid occurrence was increased (RR = 13.1, 95%CI: 1.82–29.7 and RR = 8.94, 95%CI: 1.18–59.7 respectively) [170]. Similarly, in a large retrospective study from US-based population database of electronic medical records of 62,817,650 individuals from 26 major healthcare institutions, 4530 of them were reported to have a large colonic carcinoid diagnosis and, in several cases, a personal history of CD or ulcerative colitis. For these subjects an increased risk to develop large bowel carcinoid was observed: OR 6.93 (95% CI 5.55–8.64, *p* < 0.0001) and OR 6.45 (95% CI 5.24–7.95, p < 0.0001), respectively [171]. Sciola et al. reported a prevalence for IBD of 4.8% in a series of 83 GEP NENs. This value was higher than that reported in general population [172]. In contrast, a recent Dutch nationwide study reported in the entire cohort of IBD patients from national pathological database 51 patients with concomitant IBD and colonic NENs with an estimated prevalence rate ratios between 2.8 and 4.1. These values were lower than ones from colonic resection specimens for diverticulitis and ischemia adjusted for resection type, sex and age, suggesting an incidental finding because of frequent colonic resection [173].

Detailed clinical and pathological features data for NENs in IBD is however lacking in literature. Data arose from case reports and surgical or pathological series revision with different collection data method and analysis [172, 174]. Interestingly, only a minority of patients with both diseases developed an aggressive NEN. In fact, only 8.3% (3/36) of NENs with IBD, reported by Derikx et al., showed distant metastasis (stage IV). In a recent retrospective study, Wong reported detailed clinical and pathological data in 17 patients with IBD and neuroendocrine proliferation. Eight patients (47.1%) were classified as neuroendocrine cell micronests with subcentimetric lesions and no oncological strength, while in the remain 9 (52.9%) a 1–11 mm, low grade and stage I NEN was reported [175].

### Microbiota and IBD

IBD is associated with alterations in intestinal microbiota. The pathogenesis of the disease involves complex interactions between immune system, the microbiome and environmental factors in genetically susceptible individuals [176]. Despite an impressive number of 163 genetic loci of IBD susceptibility, most of which associated with both CD and ulcerative colitis, other factors as environmental exposures seem to contribute to disease pathogenesis. Most evidences point to the interaction between the host mucosal immune system and microbes, both at the epithelial cell surface and within the gut lumen, as one of the most important factors [177]. In patients with IBD, a compositional change in microbiota and an expansion of potential pathogens have been reported. In particular, a depletion in specific commensal bacteria, as Lachnospiraceae (class of Clostridia and philum of Firmicutes) and Bacteroidetes phylum, and an enrichment in Proteobacteria was reported [178] in patients with IBD compared to healthy subjects. There are no reliable data if microbial composition changes in human have a causative role in inducing intestinal inflammation or if could be a side effect (following acute infection or host inflammatory responses) and no specific pathogenic microorganism was recognized as singular cause of chronic IBD [179, 180]. However, alterations in gut microbiota were found in CD and ulcerative colitis, with a relevant impact of aging and disease stage. In fact, a decreased amount of Roseburia hominis and *Faecalibacterium prausnitzii* (Fprau) has been observed using RT-PCR in ulcerative colitis fecal samples [181]. In CD a global decrease in the biodiversity of the fecal microbiota with markedly reduced diversity of Firmicutes and in particular of the Clostridium leptum phylogenetic group was reported using a metagenomic approach [182]. An elegant paper from Sokol reported lower proportion of Fprau in resected ileal mucosa from patients with CD associated with endoscopic recurrence at 6 months, suggesting a significant role for microbiota variation in recurrent disease [183]. In this study an anti-inflammatory property of Fprau has been demonstrated both *in vitro* and *in vivo*.

### Microbiota e NENs

Although only few studies are available in literature on microbiota and NENs, there are common patterns of microbiome composition with IBD. In 2008 Dorffel showed a significant depletion of Fprau in 12/23 patients with NENs, as previously reported in CD [181, 183]. Another study evaluated microbial fecal composition using microscopic examination and fluorescence and *in situ* hybridization in 66 patients with NENs (25 from foregut, 30 from midgut and 11 from hindgut origin), 25 healthy subjects and 50 patients with CD. Depletion of Fprau was observed in 67% of patients with midgut NENs, 84% of untreated CD and 56% of treated CD, while only in 3% of patients with chronic idiopatic diarrhea and 0% of healthy controls. In the same study fecal Enterobacteriaceae were significantly increased in NENs and CD patients. The effect of NEN therapy on microbiota was also analyzed. Somatostatin analogues had no influence on the concentration of habitual or occasional bacterial groups, while interferon alpha-2b and chemotherapy induced a massively increased in Fprau. Similar data were reported in successfully treated CD patients despite different drugs were used [184].

Therefore, a depletion of Fprau has been reported both in patients with IBD and NENs. A possible protective effect of Fprau on IBD inflammation has been proposed. Fprau is known to have a role in producing a large amount of butyrate, able to modulate the immune system and to protect the gut barrier integrity. The gut microbiota-derived butyrate, not also supplies energy source for intestinal epithelial cells, but also inhibits inflammation through epigenetic mechanisms [185]. In addition, recent data suggested a protective effect of Fprau for several tumors, such as colon carcinoma [186], breast cancer [187] and melanoma [188].

## Conclusions

In the last years there is mounting evidence supporting the role of the gut microbiome in the pathogenesis of several tumors and response to the therapy.

While HP appears to be involved in the development of gastric NENs, no clear data are currently available concerning the effect of microbiota on the development of other GEP NENs. Preliminary data reported a depletion of Fprau in patients with midgut NENs and in subjects with IBD. However, no cause-effect relationship between these events has been conclusively demonstrated.

In addition, a potential role for gut dysbiosis was reported in IBD not only for bacteria species but also for fungal microbiota (mycobiota) and viral microbiota (virobiota) [189, 190]. While, no data for mycobiota or virobiota modifications are available in NENs [191].

Further studies are required to clarify the potential role of the intestinal microbiota (including bacteria, fungi and viruses) in the development and progression of GEP NENs. These aspects could have relevant clinical implications in the prevention and therapy of these tumors.

## Abbreviations

A-CAG: Type A chronic atrophic gastritis
ATM: Ataxia-Telangiectasia Mutated protein kinase
CagA: Cytotoxin-associated gene A protein
CD: Crohn’s disease
CDK: Cyclin-dependent kinase
CDX: Homeobox protein CDX-2
c-MET: Tyrosine-protein kinase Met
COX: Cyclooxygenase
Cyc: Cyclin
DLL: Delta-like ligand
ECL: Enterochromaffin-like
EGFR: Epidermal growth factor receptor
ERK: Extracellular signal-regulated kinases
F: Factors released by HP
FOXO: Forkhead box O
FZD7: Frizzled-7
GEP: Gastroenteropancreatic
GF: Growth factor
gp30: G protein-coupled receptor 30
GSK3: Glycogen Synthase Kinase 3
HP: Helicobacter pylori
IBD: Inflammatory bowel disease
IL-6: Interleukin-6
MEK: Mitogen-activated protein kinase kinase
mTORC-1: Mammalian target of rapamycin complex 1
NBS1/NBN: Nibrin
NENs: Neuroendocrine neoplasms
NICD/CSL: Notch intracellular domain/CBF1 Suppressor of Hairless Lag1
Notch: Neurogenic locus notch homolog protein
OipA: Outer inflammatory protein A
PI3k: Phosphatidylinositol-3-Kinase
PTEN: Phosphatase and tensin homolog
RAF: Rapidly Accelerated Fibrosarcoma
RAS: Rat Sarcoma
Rb: Retinoblastoma protein
RNS: Reactive Nitrogen Species
ROS: Reactive oxygen species
RTKs: Receptor tyrosine kinases
RUNX3: Runt-related transcription factor 3
SHP2: Src homology region 2 (SH2)-containing protein tyrosine phosphatase 2
SMAD: Small Mother Against Decapentaplegic
STAT: Signal Transducer and Activator of Transcription
TFF1: Trefoil Factor 1
TGF-β: Transforming growth factor-beta
TGFR: Trasforming Growth Factor Receptor
TIVSS: Type IV secretion system
TSC2: Tuberous Sclerosis Complex 2
VacA: Vacuolating cytotoxin A
VEGFR: Vascular Endothelial Growth Factor Receptor
WNT: Wingless-related integration site
